# Retrospective analysis of the diagnostic yield of newborn drug testing

**DOI:** 10.1186/1471-2393-14-250

**Published:** 2014-07-29

**Authors:** Kelly E Wood, Lori L Sinclair, Carolyn D Rysgaard, Frederick G Strathmann, Gwendolyn A McMillin, Matthew D Krasowski

**Affiliations:** Stead Family Department of Pediatrics, University of Iowa Children’s Hospital, Iowa City, IA 52242 USA; Department of Pathology, University of Iowa Hospitals and Clinics, Iowa City, IA 52242 USA; ARUP Institute for Clinical and Experimental Pathology, Salt Lake City, Utah USA; Department of Pathology, University of Utah School of Medicine, Salt Lake City, Utah USA

**Keywords:** Meconium drug abuse detection/testing, Urine drug abuse detection/testing, Non-medical drug use, Fetal drug exposure, Prenatal drug abuse, Substance abuse testing

## Abstract

**Background:**

The objective of this study was to identify high-yield screening risk factors for detecting maternal non-medical drug use during pregnancy.

**Methods:**

A four year retrospective analysis was conducted at an academic medical center. Detailed chart review of both the newborn and mother’s medical record was performed on all cases for which one or more drug(s) or metabolite(s) were identified and confirmed in meconium or urine.

**Results:**

229 (9.2%) of 2,497 meconium samples out of 7,749 live births confirmed positive for one or more non-medical drugs. History of maternal non-medical drug and/or tobacco use in pregnancy was present in 90.8% of non-medical drug use cases. Addition of social risk factors and inadequate prenatal care increased the yield to 96.9%.

**Conclusions:**

Use of focused screening criteria based on specific maternal and social risk factors may detect many prenatal non-medical drug exposures.

**Electronic supplementary material:**

The online version of this article (doi:10.1186/1471-2393-14-250) contains supplementary material, which is available to authorized users.

## Background

Reported non-medical drug use among pregnant women in the United States is estimated at a rate of 5 per 100 births based on most recent national survey data [1]. This is likely an underestimate due to known underreporting by pregnant women [2, 3].

The lack of universal clinical indications for newborn drug testing in the United States results in variable screening practices [4]. For most institutions, universal screening of newborns for maternal non-medical drug use is impractical and not cost effective [5, 6]. Legally, providers are required to notify child protective services when a drug-exposed infant or child is identified per the Child Abuse Prevention and Treatment Act [7].

Analysis of meconium to detect fetal drug exposure has traditionally been the gold standard for newborn drug screening. Because meconium production starts around the 12th week of gestation, analysis can theoretically detect second and third trimester drug use. Disadvantages of meconium drug testing include detection of medications administered to the newborn prior to meconium collection, inconsistent distribution of analytes in the heterogeneous meconium matrix, and missed collections [4, 8].

Urine drug testing is widely used in newborn drug testing but has a short detection window capturing maternal non-medical drug use up to 3 to 7 days prior to delivery depending on the half-life of the drug [4, 9]. Dilute urine or delayed collection may result in a false negative screen even in the setting of recent maternal drug use. Like meconium, urine drug testing in newborns may pick up medications given to newborn prior to sample collection [4, 9].

Multiple maternal risk factors are associated with non-medical drug use during pregnancy which often involves multiple substances including ethanol and illicit drugs [2, 10–12]. Prenatal drug abuse may increase a mother’s risk of premature delivery, placental abruption, and precipitous delivery while also contributing to low birth weight and intrauterine growth restriction [5, 13]. Other than ethanol, data are conflicting regarding the association between maternal non-medical drug abuse with congenital malformations [5, 14].

Protocols for identifying which newborns to screen present a number of challenges for the clinical and social work team. Some of the highest risk factors for maternal non-medical drug use - such as maternal history of drug abuse, previous child protective services involvement, or domestic violence - may be difficult to elicit from the parent(s), especially if a mother presents to a facility that did not handle her prenatal care. When using meconium as the specimen for newborn drug testing, there is a risk that collection may be missed if risk factors emerge days after delivery. Thus, narrow criteria for newborn drug testing runs some risk of missing cases. On the other hand, overly broad criteria may increase sensitivity at the expense of specificity.

The objective of this study was to review newborn urine and meconium drug screening to identify high-yield screening risk factors to detect maternal non-medical drug use during pregnancy. The newborn drug testing protocol at the study institution has been used at many facilities in the state of Iowa [15].

## Methods

### Retrospective analysis at academic medical center

Retrospective analysis was conducted of the medical records of all newborns who had urine and/or meconium drug analysis studies performed over a four year period (6/2/2008 – 5/31/2012; n = 2,851) at the University of Iowa Hospitals and Clinics (UIHC). This retrospective study was Institutional Review Board approved. UIHC is a state academic medical center that serves as a tertiary care center. The medical center includes high-risk obstetric services and a level IV neonatal intensive care unit. By institutional practice, the decision to perform newborn drug screening is based on assessment of 29 items related to maternal, delivery, and newborn risk factors (see Additional file 1). The assessment tool was implemented in 2007 (prior to period of retrospective study in this report) and used throughout the entire period of retrospective analysis.

Detailed chart review was performed on all cases (n = 581) for which one or more drug(s) or metabolite(s) were identified and confirmed in meconium, and also on a randomly selected subset of 200 of the 1,916 cases (across all 4 years) without any drugs identified in meconium (random selection used “Random Sample of Cases” in SPSS, PASW Statistics 18, Chicago, IL, USA). For newborns that had only urine and not meconium drug testing performed (n = 354), all cases with positive results were reviewed in detail. Chart review included birth history (gestational age, birth weight, delivery complications), maternal history, indications for newborn drug screening, identification of prescribed medications for mother and newborn, demographics, and health insurance (summarized in Table 1). Results of meconium drug analyses performed by a national reference laboratory (ARUP Laboratories, Salt Lake City, UT, USA) over the same four year time period (6/1/2008 – 5/31/2012) were used for comparison.

**Table 1:** **Demographics, birth statistics, and health insurance status**

	Results of meconium testing			
	Group A	Group B	Group C	Group D
	No drug(s) or metabolite(s) detected ^1^(n = 1,916)	All findings explained by prescribed medication(s) (n = 283)	Non-medical drug use detected ^2^(n = 229)	Unexplained drug(s) or metabolite(s) detected (n = 69)
**Maternal age (years)** ^**3**^	26 (10)	27 (9)	26 (7)	26 (8)
**Gestational age (weeks)** ^**3**^	38 (5.2)	35.9 (7.6)	38 (5.3)	36.7 (7)
**Gravida** ^**3**^	2 (3)	2 (2)	3 (3)	3 (2)
**Para** ^**3**^	1 (2)	1 (1)	2 (2)	2 (2)
**Days meconium collected after birth** ^**3**^	0 (1)	1 (6)	0 (1)	1 (2)
***Health insurance Status***				
**Private insurance**	31.0%	30.7%	12.7%	31.9%
**Student insurance**	1.0%	0.7%	0.0%	0.0%
**Medicaid**	47.5%	42.4%	63.3%	47.8%
**Uninsured**	20.0%	25.8%	24.0%	20.3%
***Self-Declared Race***				
**Caucasian**	77.0%	72.4%	61.6%	75.4%
**African-American**	9.0%	9.5%	27.9%	10.1%
**Hispanic**	9.0%	6.0%	1.3%	5.8%
**Native American**	1.0%	1.8%	0.9%	0.0%
**Mixed race**	0.5%	1.4%	3.9%	2.9%
**Asian/Pacific Islander**	1.5%	1.4%	0.0%	0.0%
**Unknown race**	2.0%	7.4%	4.4%	5.8%

^1^Random sample of 200 within the total group of 1,916 was reviewed in detail and used for frequency calculations.

^2^Non-medical drug use includes amphetamines (amphetamine, methamphetamine, Ecstasy), benzodiazepines, cannabis, cocaine, and opioids used outside of health professional outpatient prescription or inpatient administration.

^3^Data for these variables are presented as median (interquartile range). Gravida is the number of times a woman has been pregnant. Para is the number of pregnancies carried to viable gestational age.

**Figure 1:**
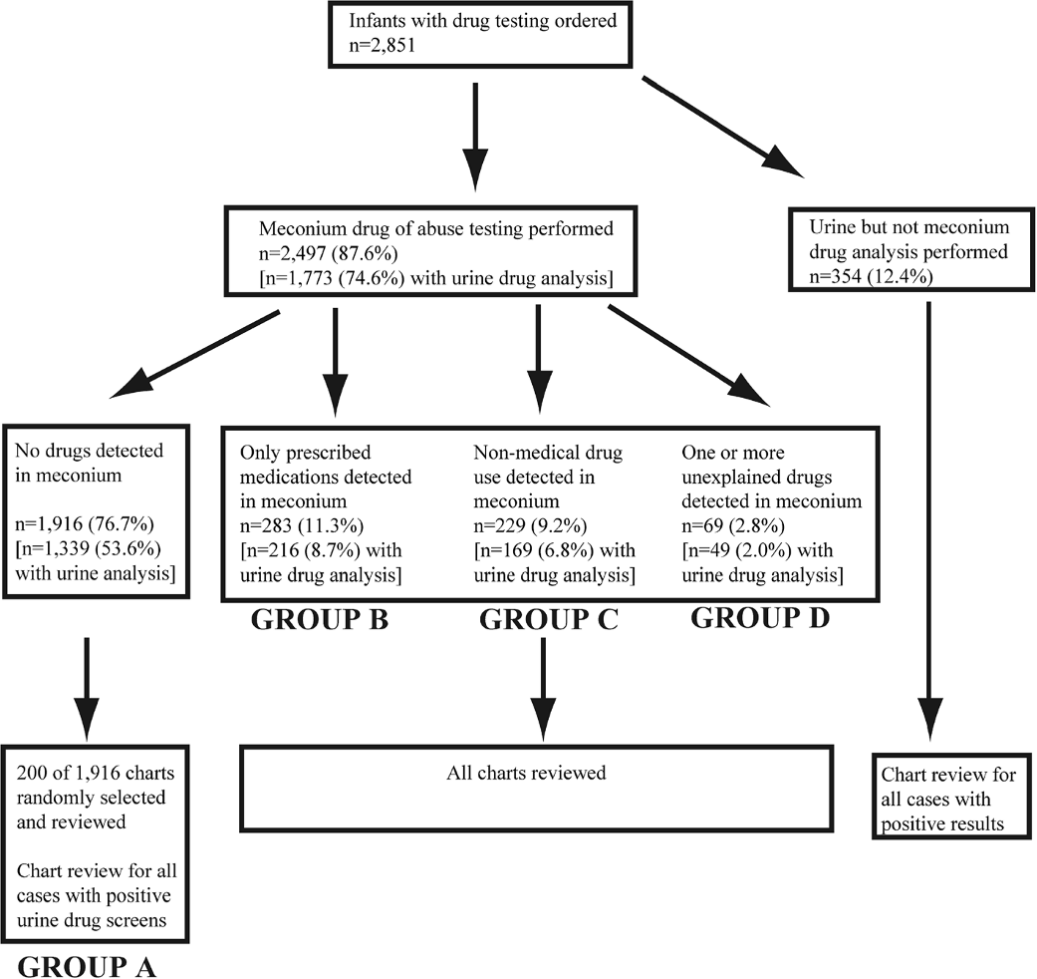
**Flow diagram of the study population categorized by results of meconium and urine drug testing.** The designation of Groups **A**, **B**, **C**, and **D** are described in detail in the Methods section.

As detailed in Figure 1 and below in the Results section, four groups were defined based on drug(s) and/or drug metabolite(s) detected by meconium drug analysis: 1. Group A-cases where testing was negative; 2. Group B-cases explained by prescribed medications given to mother and/or newborn; 3. Group C-cases where non-medical drug use was detected; 4. Group D-cases not explained by medications prescribed for mother or newborn. Categorization of drugs detected in Group C is summarized in Table 2. Group D is described in more detail in the Results section.

**Table 2:** **Categorization of non-medical drug use found in meconium**

Findings in meconium ^1^	Number of newborns
**AMPHETAMINES (n = 10, 4.4%** **)**	
Amphetamine	1
Amphetamine and hydrocodone	1
Methamphetamine +/- amphetamine^2^	6
Methamphetamine and THC^2^	2
**BENZODIAZEPINES (n = 3, 1.3%** **)**	
Alprazolam	1
Nordiazepam	1
Temazepam	1
**COCAINE (n = 16, 7%** **)**	
Cocaine	11
Cocaine and THC	5
**OPIOIDS (n = 40, 17.5%** **)**	
Codeine +/- metabolites	14
Codeine and THC	5
Hydrocodone +/- metabolites	7
Hydrocodone and oxycodone	6
Morphine +/- metabolites	3
Oxycodone	1
Oxycodone and codeine	1
Propoxyphene +/- norpropoxyphene	3
**CANNABINOIDS (n = 172, 75.1%** **)**	
THC alone	160
THC and other	12
**TOTAL NEWBORNS WITH NON-MEDICAL DRUGS**	229

^1^The data in this table encompasses both use of illicit drugs and non-medical use of prescription medications as clearly established by detailed clinical history and pharmacy review.

^2^All 8 cases that had methamphetamine also had amphetamine present (presumably as a metabolite) except for one case.

### Drug testing analysis

Meconium samples were analyzed by a reference laboratory (ARUP Laboratories) using enzyme-linked immunosorbent assay (ELISA) with confirmation by gas chromatography/mass spectrometry (GC/MS) or liquid chromatography tandem mass spectrometry (LC/MS/MS) testing [16, 17]. Results were only reported if the ELISA and confirmation methods agreed, eliminating likelihood of false positives.

Urine drug testing was performed using homogeneous immunoassays (Roche Diagnostics, Indianapolis, IN, USA) performed in the UIHC clinical laboratory. All confirmatory urine drug testing was referred to a reference laboratory (ARUP Laboratories) for analysis and quantitation by GC/MS or LC/MS/MS.

The Additional file 1 contains detailed description of analytical methods and the perinatal risk assessment tool used at the institution of study.

### Statistical analysis

Statistical analyses were carried out in SPSS. Maternal age, gestational age, gravida (number of times a woman has been pregnant), para (number of pregnancies carried to viable gestational age), and days meconium collected after birth are summarized as median and interquartile range. All other data were expressed as frequencies. The differences between Group C (non-medical drug use) and the other groups were tested by Fisher’s exact method.

## Results

### Overall rates of drug testing

During the period of study at UIHC, 7,749 live births occurred. Drug testing - urine or meconium or both - was ordered in 36.8% of live births (n = 2,851). The success rate of collecting meconium and urine was 87.6% (n = 2,497) and 74.6% (n = 1,773) respectively. Detection of non-medical drug use was reported to child protective services. Figure 1 outlines the results of testing in the study population.

Table 1 provides demographic, birth data, and health insurance status for the study populations using the four groups defined in Methods and in Figure 1.

### Meconium testing results

Of the 2,497 cases with meconium analysis, 1,916 (76.7%) were entirely negative for drug(s) and/or drug metabolite(s) (Group A). One or more drug(s) and/or drug metabolite(s) was detected in 581 (23.3%) of meconium samples.

There were 283 (11.3%) newborns for whom the compounds detected in meconium could be explained by prescribed medications given to mother and/or newborn (Group B). The most common of these medications included morphine (n = 165, 58.3%), lorazepam (n = 86, 30.4%), codeine with or without presumed metabolites such as morphine or hydrocodone (n = 41, 14.5%), and phenobarbital (n = 22, 7.8%). All positive tests for lorazepam and phenobarbital were explained by administration of prescribed medication to the newborn prior to meconium collection. Of the 165 positive tests for morphine in the absence of codeine, 157 occurred in newborns administered morphine prior to meconium collection; 8 were explained by maternal prescription for morphine, typically administration in the perinatal period. A variety of other drug(s) and/or drug metabolite(s) were detected that were consistent with documented maternal outpatient prescriptions in the 2nd and 3rd trimester including amphetamine (n = 4, 1.4%), butalbital (n = 3, 1.1%), methadone (n = 4, 1.4%), nordiazepam (a metabolite of diazepam and chlordiazepoxide; n = 2, 0.7%), and propoxyphene (n = 1, 0.4%).

Non-medical drug use was detected in 229 (9.2%) of meconium samples analyzed (Group C). Tetrahydrocannabinol (THC) (n = 172, 75.1%) was the most common drug detected. Within this group, opioids detected included codeine (n = 19, 8.3%), hydrocodone alone (n = 7, 3.1%), hydrocodone and oxycodone together (n = 6, 2.6%), morphine alone (n = 3, 1.3%), oxycodone alone (n = 1, 0.4%), oxycodone and codeine together (n = 1, 0.4%), and propoxyphene (n = 3, 1.3%). Other non-medical drugs detected include amphetamines, benzodiazepines, and cocaine (Table 2).

There were an additional 69 (2.8%) newborns who had drug(s) and/or drug metabolite(s) detected that were not explained by medications prescribed for mother or newborn (Group D); however, there was insufficient evidence to demonstrate non-medical drug use. In some cases, incomplete access to maternal medication history (often due to patients whose primary medical care was in other cities or even out of state) prevented full examination of prescription medication history. The majority of these cases (n = 58, 84%) involved various combinations of codeine with or without other opiates. The remainder were other opiates (n = 10, 14.4%) or diazepam (n = 1, 0.01%). Without clear evidence of non-medical drug use, no child protective report was pursued.

### Comparison to Meconium Drug Analysis Results from National Reference Laboratory

**Figure 2:**
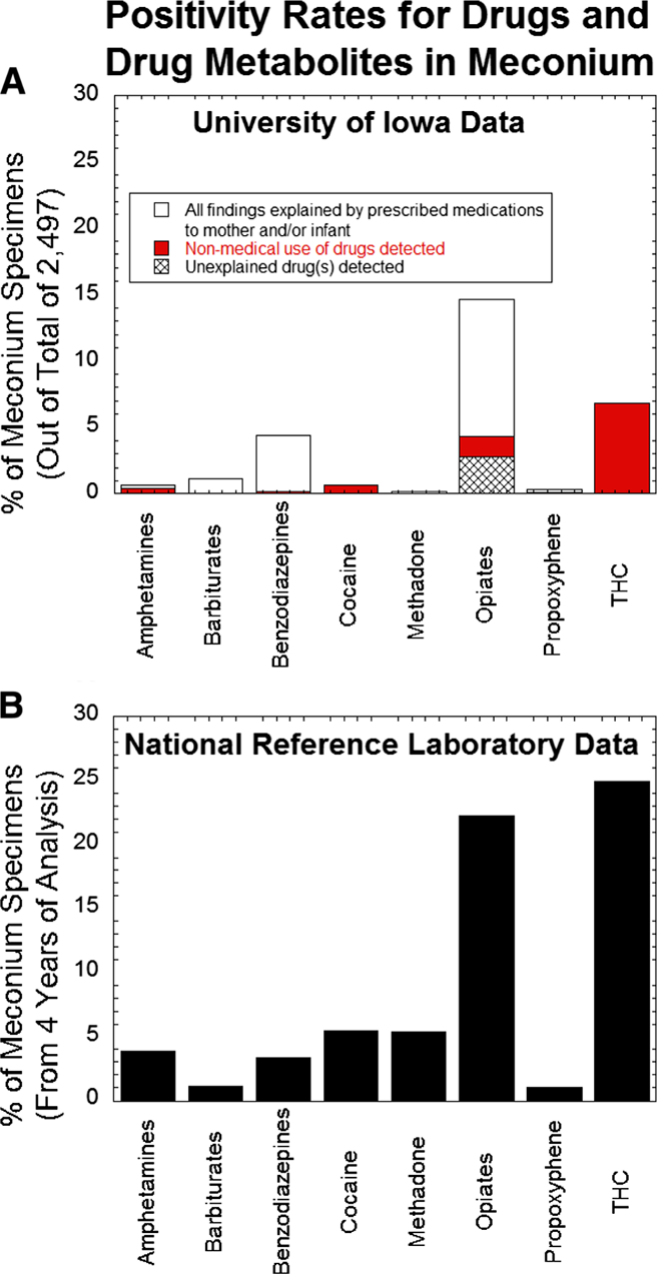
**Positivity rates for selected drugs and drug classes in meconium.** Data shown includes that observed at the University of Iowa site (panel **A**) versus de-identified data from a national reference laboratory (panel **B**). Percent positivity represents the number of unique positive specimens divided by the total number of specimens tested (not positivity rates for individual analytes).

Figure 2B shows meconium testing results over a 4 year period from a de-identified database from a national reference laboratory (ARUP Laboratories). Phencyclidine was detected in 0.1% of ARUP specimens but none of the Iowa specimens. The higher rate of positivity of barbiturates and benzodiazepines in the Iowa dataset was primarily accounted for by phenobarbital (0.9% in Iowa vs. 0.6% at ARUP) and lorazepam (3.4% in Iowa vs. 0.5% at ARUP). Given that the national reference laboratory dataset is de-identified, it is unknown what proportion of results is non-medical drug use versus prescribed medications.

### Findings in urine

Of the 2,497 newborns for whom meconium specimens were analyzed, 1,773 (71.0%) had concurrent urine testing. Only twelve urine samples (0.7%) screened positive for non-medical drug use (8 THC, 2 cocaine, 2 methamphetamine). Nine of those samples were confirmed positive by GC/MS or LC/MS/MS. The other three urine samples screened positive for THC metabolite but negative by confirmation. All twelve samples positive by screening (whether confirmed or not) demonstrated 100% concordance with meconium testing. In 128 cases where meconium analysis detected non-medical drug use, a concurrent urine specimen was negative for the drug(s) detected in meconium. There were four urine specimens that were immunoassay screen positive for amphetamines but negative by LC/MS/MS confirmatory analysis. Labetalol administration in the mother was suspected to be the likely cause of these false positives, given the reported ability of labetalol metabolites to cause amphetamine positive screens with some amphetamines immunoassays [18].

An additional 354 newborns had urine drug testing performed in the absence of meconium testing. Of these, only 14 had one or more positive results on urine drug screening. All findings were explained by medications administered to the newborn prior to sample collection except for one newborn with positive urine immunoassay screen for THC. In this newborn, there was insufficient urine specimen for confirmatory analysis, and the child abuse report was rejected.

### Yield of Screening Criteria

**Figure 3:**
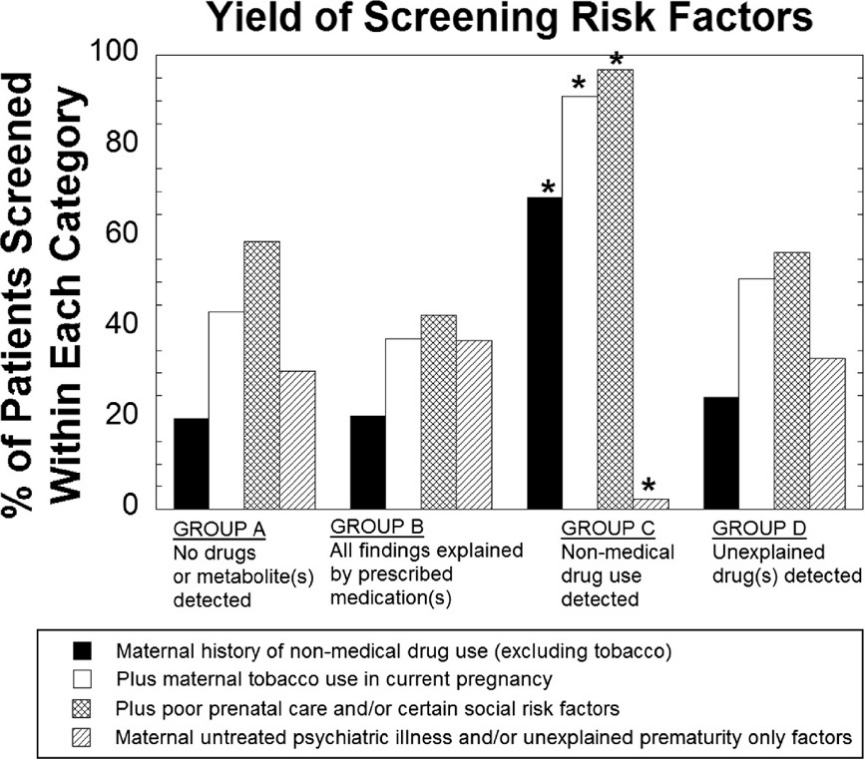
**Yield of screening risk factors for identifying maternal non-medical drug use.** History of maternal non-medical drug use, tobacco use in current pregnancy, inadequate prenatal care, and/or certain social risk factors were found in 96.9% of cases of non-medical drug use. Unexplained prematurity and/or untreated maternal psychiatric illness in the absence of other criteria were found in only 1.7% of non-medical drug use but accounted for approximately 30% of the remaining categories. *p < 0.005, Fisher’s Exact Test for comparison of Group **C** vs. Groups **A**, **B**, and **D**.

We examined how well various risk factors from the assessment tool correlated with the identification of maternal non-medical drug use (Figure 3; Table 3). A history of maternal non-medical drug use, specifically unexplained positive drug screen during pregnancy or self-report of or documented prior non-medical drug use, and tobacco use during pregnancy were significantly higher in Group C compared to the other groups (Fisher’s Exact Test p< 0.001 for all factors). The addition of poor or late prenatal care (Fisher’s Exact Test p<0.05) and/or specific social risk factors such as maternal incarceration (Fisher’s Exact Test p<0.05) were present in all but 7 of the 229 cases (96.9%) within Group C. The seven newborns (3.1%) not meeting the above risk criteria were all cases with only THC detected. Additionally, unexplained hepatitis B, hepatitis C, or human immunodeficiency virus infection (Fisher’s Exact Test p< 0.05) was significantly more common in Group C.

**Table 3:** **Most common risk factor indications for newborn drug testing**

	Results of meconium testing			
	Group A	Group B	Group C	Group D
	No drug(s) or metabolite(s) detected ^1^(n = 1,916)	All findings explained by prescribed medication(s) (n = 283)	Non-medical drug use detected ^2^(n = 229)	Unexplained drug(s) or metabolite(s) detected (n = 69)
***History of maternal non-medical drug use*** ^***2***^				
**Unexplained positive drug screen during pregnancy**	1.5%	3.2%	14.0%***	2.9%
**Maternal self-report of prior non-medical drug use**	4.5%	4.9%	27.1%***	1.4%
**Non-medical drug use in previous pregnancy**	0.5%	0.4%	3.1%**	0.0%
**Previous infant exposure to non-medical drug use**	1.0%	0.0%	0.9%	0.0%
**Documented prior history of non-medical drug use**	16.0%	15.5%	52.4%***	17.4%
**Tobacco use during current pregnancy**	33.0%	24.0%	51.1%***	36.2%
***Inadequate Prenatal care***				
**Poor (≤4 visits) or late (after week 16) prenatal care**	16.5%	5.7%	22.7%**	7.2%
***Maternal/Family Social risk factors***				
**History of domestic violence by partner**	4.5%	2.8%	6.6%	4.5%
**History of child abuse/protective services involvement**	4.0%	3.9%	5.2%	2.9%
**Incarceration**	2.0%	3.5%	6.6%*	1.4%
***Total maternal non-medical drug use excluding tobacco***	20.0%	20.5%	68.6%***	24.6%
***Total maternal non-medical drug use including tobacco***	43.5%	37.5%	90.8%***	50.7%
***Total including prenatal care and social risk factors***	59.0%	42.8%	96.9%***	56.5%
**Untreated maternal psychiatric illness** ^**3**^	22.5%	21.9%	20.5%	20.3%
**Unexplained prematurity**	26.5%	57.6%	22.3%	46.4%
***Cases screened solely due to two factors above***	30.5%	37.1%	1.7%***	26.1%
***Other factors***				
**Unexplained placental abruption**	1.0%	3.9%	0.9%	2.9%
**Unexplained maternal HBV, HCV, or HIV infection** ^**4**^	0.5%	1.8%	3.5%*	1.4%
**Unexplained infant seizures, stroke, brain infarction**	0.5%	3.9%	0.4%	2.9%
**Congenital malformations in newborn**	5.5%	14.5%	1.3%	7.2%
**Maternal age < 18 years old**	3.7%	3.8%	0.8%	2.8%

^1^Random sample of 200 within the total group of 1,916 was reviewed in detail and used for frequency calculations.

^2^Non-medical drug use includes amphetamines (amphetamine, methamphetamine, Ecstasy), benzodiazepines, cannabis, cocaine, and opioids used outside of health professional outpatient prescription or inpatient administration.

^3^This category was for untreated maternal psychiatric illness (e.g. major depression, bipolar disorder) excluding non-medical drug use.

^4^Abbreviations: HBV, hepatitis B virus; HCV, hepatitis C virus; HIV, human immunodeficiency virus.

*p < 0.05, Fisher’s Exact Test. Group C vs. Groups A, B, and D.

**p < 0.005, Fisher’s Exact Test. Group C vs. Groups A, B, and D.

***p < 0.001, Fisher’s Exact Test. Group C vs. Groups A, B, and D.

Cases for which newborn drug testing was performed solely due to the presence of either untreated maternal psychiatric illness and/or unexplained prematurity (i.e. no other risk factors present) were significantly less frequent in Group C (1.7%) versus Group A (30.5%), Group B (37.1%), or Group D (26.1%).

## Discussion

In this study the prevalence of maternal non-medical drug use per meconium drug testing at the study institution during the four year study period was 3%, which is below national estimates of 5% [1]. A large de-identified database of meconium analysis from a national reference laboratory showed higher percentages of samples positive for amphetamines, phencyclidine, propoxyphene, cocaine, opiates, and THC compared to the study institution.

We found the presence of a maternal history of non-medical drug use, tobacco use in current pregnancy, incarceration, prior child protective services involvement, domestic violence, and/or inadequate prenatal care accounted for 96.9% of cases in which non-medical drug use was detected in meconium. Maternal tobacco use combined with a history of other non-medical drug use was present in 90.8% of cases in which a non-medical drug was detected. Neither untreated maternal psychiatric illness nor unexplained prematurity in the absence of other risk factors were predictive of maternal non-medical drug use in our study. These two factors alone were the reason for screening in approximately 30% of cases.

Specific risk factors that were significantly more frequent in the cases with non-medical drug use in pregnancy included unexplained positive drug screen during pregnancy, maternal self-report of or documented prior non-medical drug use including previous pregnancies, tobacco use during current pregnancy, poor or late prenatal care, incarceration of mother, and unexplained hepatitis B, hepatitis C, and/or human immunodeficiency virus infections. It should be noted, however, that some of these risk factors were seen in a high rate of cases without drugs or drug metabolites detected in meconium. For example, within group A (no drugs or drug metabolites detected), 33.0% had tobacco use in current pregnancy, 16.5% had poor or late prenatal care, and 59.0% fell within the broad grouping of risk factors involving non-medical drug use, poor or late prenatal care, and social risk factors.

Prior studies suggest marijuana is the most common non-medical drug used during pregnancy, consistent with our data in which THC accounted for 75% of non-medical drug use [10, 19]. Although delta-9-THC, the active ingredient in marijuana, crosses the placenta, the association of prenatal marijuana use with premature delivery, low birth weight, or congenital malformations is not clear [3, 5, 20–23]. Long term, prenatal marijuana exposure may have adverse effects on learning, attention and behavior [5]. Three urine specimens in our study screened positive for THC but showed negative confirmatory testing, a phenomenon previously noted in other studies [24, 25].

Opiate exposure is estimated to occur in 2 to 20% of pregnancies [26]. Recent studies using meconium drug testing have documented misuse of prescription pain medication among pregnant women [27, 28]. The incidence of non-medical use of prescription opiates identified via meconium drug testing was 0.5% (n = 40) in this study. This figure excludes the 68 unexplained positive meconium samples for opiates. Interpretation of positive opiate screens is complicated given the complexity of opiate metabolism and multiple possible sources of opiates such as medications prescribed for the mother and/or newborn, poppy seeds, heroin, or intentional misuse of prescription opiates (See Additional file 1) [9].

A major finding of this study is that detection of prescribed medications is common with meconium and/or urine drug screening. The study institution includes a neonatal intensive care unit which cares for many premature infants. Many premature infants receive medications prior to the passage of meconium, which may be delayed until the ninth day of life [28]. Morphine, lorazepam, and phenobarbital administered to newborns prior to meconium collection accounted for 96.5% of the 283 samples whose meconium findings were completely explained by prescribed medications. Detection of prescribed medications requires thorough review of the maternal and newborn medical records, along with consideration of the metabolic pathways of opiates and benzodiazepines, to avoid unnecessary accusations of non-medical drug use (See Additional file 1).

Our study found very low yield of urine drug screening. Over a 4 year period, urine drug screening did not detect any non-medical drug use not seen in meconium. In addition, urine drug testing failed to detect 128 cases of non-medical drug use determined by meconium analysis.

Study limitations include analysis of a single academic site with a primarily Caucasian patient population and incomplete data for chart review for some patients. Only newborns meeting protocol criteria had screening ordered (63.2% of the live births in the University of Iowa sample did not meet screening criteria). Some risk criteria for newborn drug screening were dependent on maternal self-reporting of data. Incarceration was not a formal screening criterion per the study institution’s protocol and only identified if specifically mentioned in the clinical documentation. The protocol used was specific to the institution. False negative tests may have occurred if sample drug concentrations fell below testing cutoffs. In addition, maternal non-medical drug use may have been missed in some cases if there was both prescription and non-medical use of the same drug or drugs sharing common metabolites during pregnancy (e.g. non-medical use of morphine by mother in pregnancy but administration of prescription morphine to the newborn prior to meconium collection). Specimens were not collected on all newborns with drug testing ordered, a known challenge given the logistic challenges with performing wide scale newborn drug testing.

Future studies using umbilical cord for newborn drug testing would be of interest to compare with the findings in meconium. Unlike meconium, universal collection of umbilical cord specimens at birth is feasible. Specimens can be held upwards of 2 weeks, allowing for newborn drug testing to occur even when risk factors emerge days after birth. Use of umbilical cord would also avoid detection of newborn medications, which in our population was a very common finding.

## Conclusions

Overall, our study demonstrates that maternal history of non-medical drug use and tobacco use in pregnancy were the highest yield risk factors for identifying non-medical drugs in meconium. Inadequate prenatal care and social risk factors were also helpful. Newborn urine drug testing was poor for detecting maternal non-medical drug use and has little diagnostic yield. Meconium drug testing frequently detects prescribed medications, necessitating a thorough review of the pharmacy history. Our results suggest that focused screening criteria based on specific maternal risk factors may detect many prenatal non-medical drug exposures.

## Electronic supplementary material

Additional file 1: **Microsoft Word document with detailed description of analytical methods, the perinatal risk assessment tool used at the institution of study, and metabolic pathways of opiates and benzodiazepines.** (DOCX 1 MB)

## Abbreviations

ELISA: Enzyme-linked immunosorbent assay
GC/MS: Gas chromatography/mass spectrometry
LC-MS/MS: Liquid chromatography tandem mass spectrometry
THC: Tetrahydrocannabinol
UIHC: University of Iowa Hospital and Clinics
HBV: Hepatitis B virus
HCV: Hepatitis C virus
HIV: Human immunodeficiency virus.
