# Supplementary material for: Retrospective analysis of the diagnostic yield of newborn drug testing
Source: BMC Pregnancy Childbirth. 2014 Jul 29;14:250. doi: 10.1186/1471-2393-14-250 (PMC4124162; doi:10.1186/1471-2393-14-250)
Supplement: Supplementary file 1 — Additional file 1: Microsoft Word document with detailed description of analytical methods, the perinatal risk assessment tool used at the institution of study, and metabolic pathways of opiates and benzodiazepines. (DOCX 1 MB) [file 12884_2013_1121_MOESM1_ESM.docx]

**Additional File 1**

**Text summary:** The Additional File contains detailed description of analytical methods, the perinatal risk assessment tool used at the institution of study, and metabolic pathways of opiates and benzodiazepines.

**Additional Methods**

*Drug Testing Analysis*

The meconium testing protocol used was a nine-drug panel that included amphetamines [amphetamine, methamphetamine, methylenedioxymethamphetamine (MDMA-Ecstasy), methylenedioxyethylamphetamine (MDEA-Eve), and methylenedioxyamphetamine (MDA)], barbiturates (amobarbital, butalbital, pentobarbital, phenobarbital, and secobarbital), benzodiazepines (alprazolam, α-hydroxyalprazolam, clonazepam, 7-aminoclonazepam, desalkylflurazepam, 2-hydroxyethylflurazepam, diazepam, lorazepam, midazolam, nordiazepam, oxazepam, temazepam, and α-hydroxytriazolam), cocaine (cocaine, benzoylecgonine, *m*-hydroxybenzoylecgonine), methadone [(methadone and 2-ethylidene-1,5-dimethyl-3,3,-diphenylpyrrolidine (EDDP)], opiates (codeine, morphine, 6-acetylmorphine, dihydrocodeine, hydrocodone, hydromorphone, oxycodone, and oxymorphone), phencyclidine, propoxyphene (propoxyphene and norpropoxyphene), and tetrahydrocannabinol (THC; 9-carboxy-THC or in some cases 11-hydroxy-THC as well).

Urine drug testing was performed using homogeneous immunoassays (Roche Diagnostics, Indianapolis, IN, USA) performed in the hospital clinical laboratory. At the start of the period of retrospective analysis, the routine urine drug of abuse panel consisted of six tests: amphetamines, barbiturates, benzodiazepines, cocaine metabolite (benzoylecgonine), opiates, and THC metabolite (9-carboxy-THC). Approximately midway through the period of retrospective analysis due to change in institutional procedure, the panel was changed by dropping the barbiturates screen, adding an oxycodone/oxymorphone screen, and separating out THC screening as a separate test. Throughout the period of retrospective study, clinicians could order urine drug screening with or without reflex confirmation for amphetamines, barbiturates, cocaine, and THC. Due to the frequency of inpatient use of benzodiazepines and opiates, confirmatory testing for these two classes of drugs requires additional provider order. All confirmatory urine drug testing was referred to a reference laboratory (ARUP Laboratories) for analysis and quantitation by GC/MS or LC/MS/MS.

*Opiate and Benzodiazepine Metabolism Pathways*

Opiates and benzodiazepines have complicated overlapping metabolism pathways as shown below.


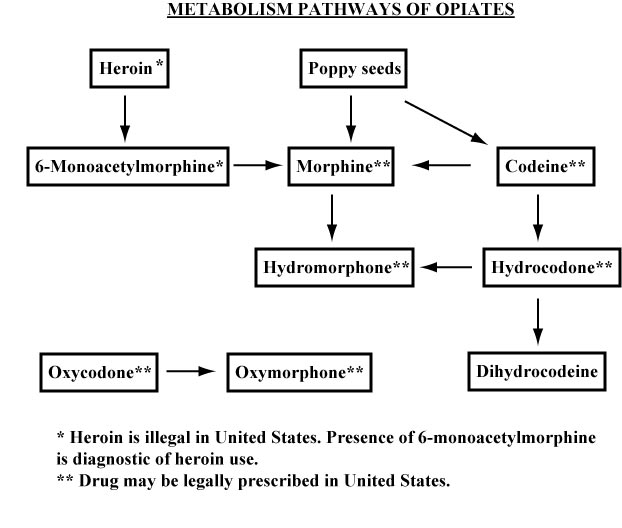


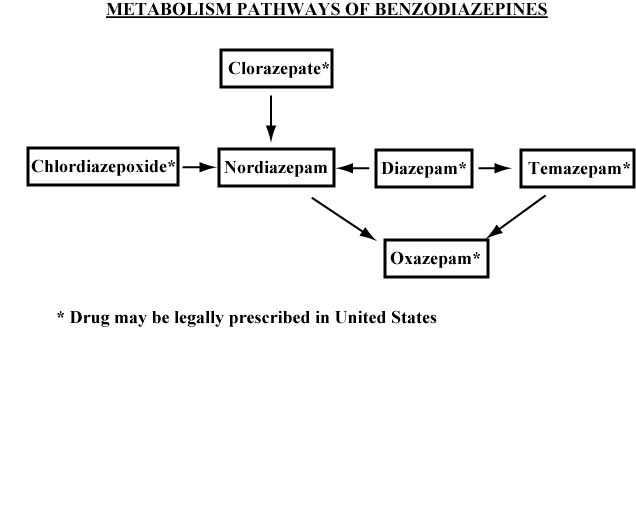


***Perinatal Illicit Drug/Exposure Risk Assessment Tool Used at University of Iowa Hospitals and Clinics (UIHC)***

**Any single positive factor from the list below initiates urine and meconium drug testing of newborn.**

**Risk Factors Related to History of Maternal Non-Medical Drug Use**

• Maternal urine drug screen positive

• Maternal self report of illicit drug use

• Tobacco use during pregnancy

• Physical attributes in mother suggesting illicit drug use such as IV track marks,

visible tooth decay, sores on face, arms, or legs

• Altered mental status suggesting influence/withdrawal from illicit drugs

• Mother ever used illegal drugs during previous pregnancies

• Mother previously delivered an infant who tested positive for illicit drugs

• Documented history of illicit drug use by mother within the last 3 years

• History of illicit drug rehabilitation by mother within the last 3 years

**Risk Factors Related to Maternal/Family Social History**

• History of domestic violence by partner within the last 3 years

• History of prior Department of Human Services (DHS) involvement due to child abuse, neglect, or court ordered placement of children outside of home of parents

**Risk Factors Related to Inadequate Prenatal Care**

• No prenatal care or late prenatal care (> 16 weeks gestation)

• Unexplained poor prenatal care (≤ 4 prenatal visits)

• Unexplained discrepancy between delivery/prenatal care facilities (hospital hopping)

**Risk Factors Related to Medical Complications in Pregnancy**

• Unexplained placental abruption

• Unanticipated out-of-hospital delivery

• Unexplained presentation at hospital in second stage of labor

• Unexplained precipitous labor (<3 hours)

• Unexplained episode of acute hypertension (≥ 140/90 mmHg)

• Unexplained seizures, stroke, or myocardial infarction

• Unexplained stillbirth

**Risk Factors Related to Maternal Medical History**

• Unexplained Hepatitis B or C virus, syphilis, or human immunodeficiency virus (HIV) infection within the last 3 years

• Untreated maternal depression or major psychiatric illness within the last 3 years

**Risk Factors Related to Newborn Assessment**

• Gestation < 37 weeks from unexplained preterm delivery

• Unexplained newborn birth weight less than 10^th^ percentile for gestational age

• Unexplained newborn head circumference less than 10^th^ percentile for gestational age

• Unexplained newborn seizures, stroke, or brain infarction

• Unexplained newborn symptoms that may suggest drug withdrawal/intoxication: high

pitched cry, irritability, hypertonia, lethargy, disorganized sleep, sneezing,

hiccoughs, drooling, diarrhea, feeding problems, or respiratory distress

• Unexplained newborn congenital malformations involving genitourinary tract,

abdominal wall, or gastrointestinal systems
